# To be or not to be relevant: Comparing short- and long-term consequences across working memory prioritization procedures

**DOI:** 10.3758/s13414-023-02706-4

**Published:** 2023-05-01

**Authors:** Stephanie Jeanneret, Lea M. Bartsch, Evie Vergauwe

**Affiliations:** 1 Faculty of Psychology and Educational Sciences, University of Geneva, Geneva, Switzerland; 2 Department of Psychology, University of Zurich, Zürich, Switzerland

**Keywords:** Working memory, Prioritization, Long-term memory, Attention, Focus of attention

## Abstract

Priority-based allocation of attentional resources has shown robust effects in working memory (WM) with both cue-based and reward-based prioritization. However, direct comparisons between these effects in WM are needed. Additionally, the consequences of WM prioritization for remembering in the long term remain unclear for both prioritization procedures. Here, we tested and compared the immediate and long-term memory (LTM) effects of cue-based versus reward-based retrospective prioritization of WM content. Participants encoded four memory items and were then indicated to prioritize one of the items through the presentation of either a retro-cue or a reward pattern. We then tested their immediate and delayed memory. The results of the first experiment showed better memory for prioritized than for unprioritized information in WM and LTM, but the WM effect was driven solely by the retro-cue, making it difficult to interpret any reward-based effects in LTM. In the second experiment, using a more explicit and meaningful reward-based manipulation, the results showed a prioritization benefit in WM for both prioritization procedures. In LTM, however, the prioritization effect was predominantly driven by the retro-cue manipulation. Taken together, we found that (1) the way in which attention is directed in WM impacts the size of the prioritization benefit in WM, (2) WM prioritization generally results in a prioritization effect in LTM, and (3) that the effect in LTM is more robust for cue-based prioritization. Exploratory analyses indicated that the LTM effect of cue-based prioritization reflected a cost in performance for noncued items rather than a benefit for cued items.

**Supplementary Information:**

The online version contains supplementary material available at 10.3758/s13414-023-02706-4.

## Significance

Although an important human ability is to hold multiple pieces of information in mind at the same time, we often need to selectively focus on some information at a given instance. Prioritizing behaviorally relevant information in memory is a crucial cognitive ability and is assumed to have implications for short-term and long-term memory. Yet the precise ways in which we prioritize information have not been directly compared, and the consequences for long-term memory are currently unclear. This work not only provides insight into basic working memory processes but also brings together procedures that have largely been studied separately, and hence will further our understanding of human memory.

## Introduction

In our daily lives, we often need to juggle memories to fulfill a given task, concentrating on some while keeping others in the back of our mind. The act of holding certain pieces of information temporarily at the “forefront” is a hallmark of working memory (WM). Much work has focused on understanding the role that attention plays in manipulating information in WM to help us selectively focus on behaviorally relevant content. Several researchers have proposed the existence of distinct, priority-dependent WM representational states, whereby certain memories are qualitatively distinct from others at a given instance (Muhle-Karbe et al., 2021; Nee & Jonides, 2013; Olivers et al., 2011; van Moorselaar et al., 2015).

Recent behavioral and neural models highlight the notion of such representational states in WM. For example, the three-state model (Oberauer, 2002) based on the embedded-processes model of Cowan (1999) describes how an attentional spotlight gives access to distinct pieces of information in WM in a hierarchical framework that goes from a passive to an active state: the *activated part of LTM* contains relevant information, from which a subset is taken into a *region of direct access*, and further information is retained inside the *focus of attention*. In this model, the focus of attention contains a single, high-priority item that benefits from a privileged status in WM (Garavan, 1998; McElree, 2006; Oberauer & Hein, 2012).

Neural and computational evidence has shed light on the possible mechanisms that produce distinct WM states (Jonides et al., 2008; Manohar et al., 2019; Muhle-Karbe et al., 2021; Nee & Jonides, 2013; Rose, 2020; Stokes et al., 2020). These mechanisms may be considered to be additive in nature with one type of activity instantiating another: activity-silent mechanisms mediated by synaptic plasticity sustain the activated part of LTM; a subset of those sustained memories is retrieved into a region of direct access via context bindings mediated by the hippocampus; and active neuronal firing selects items inside the focus of attention, which may lead to enhanced neural patterns (Chun, 2011; LaRocque et al., 2014). Synaptic plasticity may also passively maintain latent representations, and further influence cognition and memory (Manohar et al., 2019; Rose, 2020; Stokes et al., 2020). Nevertheless, both sustained activity and activity-silent mechanisms (i.e., synaptic plasticity) appear to serve memory and attention. Much work has been dedicated to investigating the behavioral implications of selecting memories into the focus of attention due to prioritization in WM and shifting the focus of attention to prioritize certain memories, which we will review next.

### Cue-based and reward-based prioritization benefits in WM

Recent literature has elucidated multiple ways that WM content can be prioritized, which are typically cue- and reward-based experimental paradigms of directing attention in WM (Atkinson et al., 2018). In the most common cue-based paradigm, retrospectively guided attention to an item via a *retro-cue* improves memory for that item at test, compared with memory for baseline items which were not cued in WM (i.e., the retro-cue effect; Pertzov et al., 2017; Souza & Oberauer, 2016 for a review). In a recent reward-based (value-based) paradigm, sequentially presented items associated retrospectively with relatively higher probe values were better recalled at test (Allen & Atkinson, 2021). That is, arbitrary point values (and not monetary values) such as “1” and “4” were assigned to different items, and these reflected “low” and “high” point rewards, respectively.

Even though both paradigms involve the prioritization of information within WM, resulting in improved immediate recall performance for the prioritized information, it is unclear whether the same mechanisms support these effects in both procedures. The retro-cue effect in WM has been explained by several mechanisms, including the freeing up of capacity and of attentional resources (see Souza & Oberauer, 2016, for a review of hypotheses supporting the retro-cue effect). This allows for cued items to be prioritized and reside inside the focus of attention, resulting in better WM performance for cued items, compared with performance for baseline items (Souza & Oberauer, 2016). Reward-based prioritization benefits have been explained by a strategic allocation of attentional resources. This strategic prioritization allows high-reward items to be more accessible inside the focus of attention (Allen & Ueno, 2018; Hu et al., 2014), or refreshed more frequently or for a longer period of time than low-reward items are, resulting in better WM performance for high-reward items (Allen & Atkinson, 2021).

It is important to note differences between both prioritization procedures regarding the validity of the priority signal. In the typical retro-cue paradigm, the prioritized item is always tested (i.e., 100% valid cue). Thus, in the retro-cue paradigm, the prioritized item is the *only relevant* item and the other items in WM can be considered *irrelevant*. In the reward-based paradigm, a single item is associated with the greatest probe value, but all items may nonetheless be tested. Thus, in the reward-based paradigm, the prioritized item is *more relevant* and the remaining items in WM are *less relevant*. It is possible that this relevance-based difference between both procedures results in differences in their respective impact on immediate recall performance.

It is currently unclear how WM prioritization effects are scaled when manipulated within a single experiment. Therefore, the first aim of our study was to provide a direct comparison of the prioritization benefits in WM between cue- and reward-based prioritization procedures. Further, given the theoretical frameworks that describe the focus of attention as being part of activated LTM (Cowan, 1999; Oberauer, 2002), and the neural and behavioral evidence positing the contribution of WM maintenance for LTM (Ranganath et al., 2005), WM prioritization effects should reasonably extend into LTM.

### Opposing hypotheses on the long-term consequences of WM prioritization

There are at least two possible ways that WM prioritization can impact LTM. One hypothesis postulates a long-term prioritization boost, whereby prioritized information gets strengthened in LTM, leading to similar beneficial effects of prioritization as those observed in WM. According to the information processing account, the way in which information is processed in WM modulates how well it can be retrieved from LTM later on (Craik & Watkins, 1973), and it has been argued that focused attention on WM content improves LTM for the information in question (e.g., Souza & Oberauer, 2017). Accordingly, bidirectional interactions of attention and LTM have shown that the formation of episodic memories is strongly dependent on how much time attention is devoted to memoranda (Hannula, 2018).

To our knowledge, three studies have shown support for the long-term prioritization boost hypothesis. Reaves et al. (2016) observed cuing effects in LTM and show neural evidence reflecting spatial attention^1^ that predicted subsequent LTM performance. In this task, both retrospective and prospective prioritization were used: a spatial pre-cue first indicated which side of the screen would be probed before the simultaneous memory item presentation, which was then followed by the retro-cue. A similar study has also shown lasting retro-cue effects in both younger and older adults, and proposed that cued items are encoded into richer LTM traces, compared with their noncued counterpart (Strunk et al., 2018). Additionally, Sandry et al. (2020) observed a behavioral benefit of reward-based prospective prioritization of sequentially presented verbal memory items on a surprise LTM test. Here, prioritized items were presented in red and were worth 25 points, whereas unprioritized items were presented in black and were worth 3 points.^2^ Delayed recall performance was greater for prioritized items than it was for unprioritized and baseline items. As mentioned earlier, this prioritization benefit of sequentially encoded items can be explained by attentional refreshing, a mechanism which may lead to improved LTM (Johnson et al., 2002).

This long-term reward-based effect is further supported by literature examining the influence of monetary reward incentives in LTM. For example, there is neuroimaging evidence that reward-related, dopaminergic brain activity promotes or predicts subsequent memory (Adcock et al., 2006; Gruber & Otten, 2010; Wittmann et al., 2005). To illustrate, functional MRI activation of dopaminergic midbrain regions, such as the substantia nigra (Wittmann et al., 2005) and the ventral tegmental area (Adcock et al., 2006), has been associated with enhanced LTM. Further, electrical brain activity associated with a monetary reward has been shown to be predictive of subsequent memory when the reward corresponded to a high-reward cue (Gruber & Otten, 2010). Collectively, these studies suggest that rewards not only elicit greater activation in dopaminergic and hippocampal brain areas, but also boost memory formation.

According to a second hypothesis, a long-term impairment of prioritization, WM prioritization impairs LTM performance for prioritized information (LaRocque et al., 2015). The argument is that if an item is not in the focus of attention, it is reinstated as a LTM trace. Therefore, LTM-dependent delayed recall should be better for unprioritized representations. This hypothesis stems from neural decoding evidence showing that brain signals for unprioritized (unattended) information can be reactivated (Lewis-Peacock et al., 2012; Rose et al., 2016), and that this information is better recovered than prioritized information is following distraction (Mallett & Lewis-Peacock, 2019). Others have also shown that the signal for unprioritized items remained relatively higher throughout the maintenance period when objects (vs. features) were behaviorally relevant to the task (Sahan et al., 2020). Despite these findings in WM, LaRocque et al. (2015) tested but did not find support for a long-term impairment (or boost) of prioritization. In their study, no difference was found in LTM performance between prioritized and unprioritized information.

Given the current state of the literature, the second aim of our study was to clarify our understanding of the long-term consequences of WM prioritization. Moreover, it is also not known whether the long-term consequences of WM prioritization depend on *how* information was prioritized in WM. Thus, the third aim of our study was to directly compare any LTM effects of cue- and reward-based prioritization procedures.

### The present study

In this study, our goals were three-fold: we aimed to (1) compare the short-term effects of cue- and reward-based retrospective prioritization procedures in WM, (2) assess the overall long-term effects of WM prioritization, and (3) compare these long-term effects with respect to the type of WM prioritization. We predicted to observe prioritization benefits in WM and LTM, whereby prioritized information gets boosted in memory. Due to the variation in the degree of relevance of items across cue- and reward-based procedures from relevant to irrelevant (cue-based) and more or less relevant (reward-based), we also hypothesized that the WM and LTM prioritization benefits would be more pronounced for retro-cued than for high-reward items.

## Method

### Participants

Sixty-two (62) undergraduate psychology students (Experiment 1: eight male, 21 female; mean age = 26.21 years; Experiment 2: seven male, 26 female; mean age = 21.67 years) from the University of Geneva^3^ participated for partial course credit. We chose the initial sample size of *n* = 30 for both experiments because we assumed it would be sufficient to detect the effects of interest in our within-subjects design. The use of Bayesian statistics means that the sample size can be increased in case of ambiguous evidence until the ambiguity is resolved (see Rouder, 2014, for optional stopping in Bayesian statistics). We sampled 33 participants for Experiment 2, to account for potential to-be-excluded subjects (as we had three in Experiment 1). Both experiments were conducted following approval from the Ethical Committee at the University of Geneva. Each participant provided written informed consent.

### Materials and stimuli

Experiments 1 and 2 were conducted in the lab. Experiment 1 was presented on Dell monitors with participants run in groups of six. Due to COVID-19 restrictions, we ran Experiment 2 one participant at a time, on a Dell laptop. Both experiments were run with MATLAB software and the Psychophysics Toolbox (Version 3; Brainard, 1997; Kleiner et al., 2007; Pelli, 1997). The stimuli used in both experiments originated from the database shared by Brady et al. (2008) and were drawn from a pool of 2,402 object images.

### Design

**Fig. 1:**
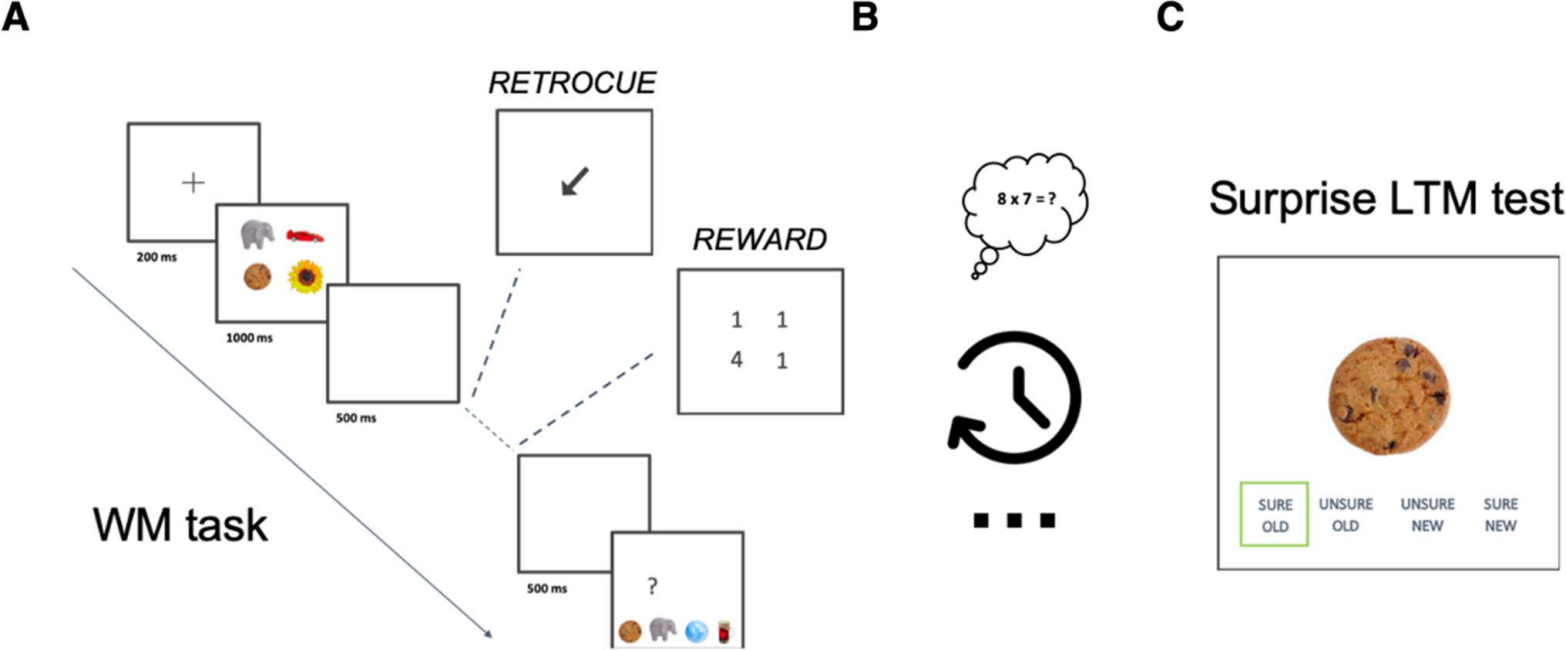
Illustration of the study paradigm. *Note.* Participants began with a four-choice probed recognition WM task (**A**), followed by a filler task (**B**), and ended with the surprise four-choice recognition LTM test (**C**). (Color figure online)

Participants first completed a probed recognition WM task (see Fig. 1), in which they encoded four items simultaneously presented on screen for 1,000 ms at four locations. Critically, after a 500-ms blank screen, participants were then presented with either a retrospective cue (retro-cue) or a 1-1-1-4-point reward pattern (reward). Following another 500-ms blank screen, participants’ memory for one of the items of the trial was tested by presenting a question mark at one of the four locations on screen. Participants were instructed to select the item that had appeared at that location by choosing among four response options at the bottom of the screen. Trials were self-paced, so the test window remained on screen until the participant clicked on an item with the computer mouse. The response options included: the tested item (e.g., *cookie*), a within-trial lure (i.e., another item from the same trial as the target item, e.g., *elephant*), a previous-trial lure (an item randomly picked from a previous trial, e.g., *ball*), and a never-presented lure (a new item that was never presented before, e.g., *beer glass*).

Following the WM task, participants completed a two-minute filler task in which they responded to as many arithmetic problems as possible. Finally, participants completed a surprise old/new recognition test, where we tested incidental LTM for WM items. On each trial, one item was first presented for 500 ms before allowing for the participant to respond whether it was old or new and rate their confidence for that judgment.^4^ Participants chose from the following response options: “sure OLD,” “unsure OLD,” “unsure NEW,” “sure NEW” (Fig. 1). As in the WM task, responses were self-paced. Feedback was provided after each trial response (a correct response was boxed in green; an incorrect response was boxed in red).

Both retrospective prioritization procedures used in the WM task are described next. Importantly, there are many ways to implement these procedures; for optimal comparison, we used rather classical implementations of cue- and reward-based paradigms in visual WM. For the retro-cue condition, an arrow pointing to the location of a previously encoded item instructed participants to guide their attention to that item in WM (Souza & Oberauer, 2016). Critically, the retro-cue was 100% valid. Thus, the to-be-tested item was always the *prioritized* item, whereas all other items in the WM set were *unprioritized* items. Additionally, we included two experimental controls for retro-cue trials. As is state of the art in the literature (Rerko et al., 2014), we presented baseline trials in which an empty delay followed item presentation, in lieu of the retro-cue. Baseline trials allowed us to test WM for *unprioritized* items. We also presented no-test trials, in which a retro-cue pointed to one of the locations during an empty delay in lieu of the test window. These trials were randomly intermixed with retro-cue trials and allowed us to assess subsequent memory for prioritized items that were not tested in WM (*prioritized *and* untested* items), thereby controlling for testing effects in LTM for prioritized items. For the reward condition, we used value-based prioritization, retrospectively (Allen & Atkinson, 2021). The reward associated one of the previously presented items in the WM set with four (4) points (the *prioritized* item) and all three other items with one (1) point (the *unprioritized* items). Critically, each item was tested with equal probability (25%). Thus, both prioritized and unprioritized items were tested in WM.

In the WM task, we presented 144 trials (4 items per trial, 576 items in total), systematically distributed across three blocks as follows: 32 trials in the retro-cue block; 16 no-test trials randomly intermixed in the retro-cue block; 32 baseline trials in a separate block; and 64 trials in the reward block. The order of the three blocks was counterbalanced across participants. Within each block, there were breaks every 10 minutes. In the subsequent surprise LTM test, we presented 320 trials consisting of old and new items. One-half of the trials presented old items from the WM task; of those trials, we tested items from each prioritization condition evenly. Thus, 64 items were randomly drawn from reward trials (half of which were prioritized items, and in each half evenly split between tested and untested items); 64 items were randomly drawn from retro-cue trials (half of which were prioritized items); and 32 items came from baseline trials^5^. The total task duration was approximately 20 minutes for the WM task and 30-35 minutes for the LTM test. Including nine WM practice trials, the filler task, and task debriefing, total experiment runtime was about one hour.

The design of Experiment 2 was identical to that of Experiment 1, except for two changes. First, to increase the sensitivity of the LTM test, we additionally presented exemplar-specific target lures in the delayed recognition task, so that participants had to discriminate between old items (targets, e.g., the *cookie*), target lures (e.g., a picture of a different cookie), and novel items (as in Experiment 1, items never seen before). Second, we adapted the reward manipulation, following the lack of evidence for the prioritization benefit via the reward in WM and LTM (see Experiment 1 Results). Specifically, we modified the instructions for the reward condition, such that participants were informed that for every correct response, they would earn 1 or 4 points, depending on whether a high-reward or low-reward item was tested. They were given feedback with the number of points they gained every 10 trials, and at the very end of the experiment, were told how many total points they had collected in total. They were also told they would win a small prize at the end of the experiment.^6^ The accumulated points were displayed at the end of each mini block in the reward block of the WM task. At the end, all participants received a pen or pencil of their choice, regardless of the number of points they accumulated in the reward block. Here, our goal was to assess whether the weak impact of the reward-based prioritization would be remediated with a more explicit and meaningful reward pattern manipulation, and hence allow for a better evaluation of its implications in LTM. Experiment 2 was analyzed in the same way as Experiment 1.

### Hypotheses and comparisons

In both Experiments 1 and 2, we implemented a 2 × 2 within-subjects design, with *prioritization type* (retro-cue vs. reward conditions) and *prioritization status* (prioritized vs. unprioritized items) as experimental factors in WM. We predicted that both WM and LTM would benefit from prioritization, whereby prioritized information gets boosted in memory. Specifically, for the retro-cue condition, we hypothesized that in WM, memory for cued items would be greater than baseline items; in LTM, memory for cued items would be greater than baseline items and noncued items. For the reward condition, we hypothesized that memory for high-reward items would be greater than low-reward items in both WM and LTM. Due to the variation in the degree of relevance of items across cue- and reward-based procedures from relevant to irrelevant (cue-based) and more or less relevant (reward-based), we also hypothesized that the WM and LTM prioritization benefits would be more pronounced for cued than for high-reward items.

In both cases of cue- and reward-based prioritization, one can assume that more attentional focusing during WM maintenance not only leads to improved WM for cued and high-reward items, but that this increased attentional focusing may also lead to a stronger encoding of information into LTM. Indeed, as we explained in our introduction, a common assumption is that information that gets attended to more, is more likely to be encoded strongly into LTM. In the case of retro-cuing, one could assume that the cued item is the only item that is receiving focused attention; in the case of reward-based prioritization, one could assume that all items receive focused attention, but the high-reward item receives more of it. Under these assumptions, in both cases, prioritized information receives more attention than unprioritized information and thus, prioritized information is likely to be more strongly encoded into LTM. Finally, since cued items receive the most attention compared with high-reward items, cued items might benefit from prioritization even more so than high-reward items do.

### Data preparation

Participants who performed at or under the guessing rates of both WM (25%) and LTM tasks (50%) were excluded from data analysis. In Experiments 1, three participants performed below chance-level accuracy; a total of *N* = 27 were kept for analysis. In Experiment 2, one participant performed below chance-level accuracy; a total of *N* = 32 were kept for analysis.

In WM, correct responses were defined as the binary responses for selecting the correct target item in a WM trial and were measured as a function of prioritization type for each subject. In LTM, correct responses were measured as a function of an item’s state (“old” = from WM, or “new” = novel item). Hits occurred when a participant correctly reported an old item (“old SURE” or “old UNSURE” responses to old items), and correct rejections occurred when a participant correctly reported a new item (“new SURE” or “new UNSURE” responses to new items). Misses occurred when a participant missed reporting an old item (“new SURE” or “new UNSURE” responses to old items), and false alarms occurred when a participant reported an old item that was new (“old SURE” or “old UNSURE” responses to new items). High-confidence hit rates were calculated as the mean response scores of only “old SURE”. As an index of LTM accuracy, *d’* signal detection scores were calculated using the *psycho* R package (Makowski, 2018), to obtain a measure of the distance between the signal (high-confidence hits and correct rejections) and signal + noise (misses and false alarms; Banks, 1970).

## Results

### Bayesian analyses

We analyzed the data using Bayesian mixed effect models (LME) using the *lmBF* function implemented in the *BayesFactor* package (Morey et al., 2015; Morey & Rouder, 2018) in R (R Core Team, 2017). For the WM data, the dependent variable of interest was the mean proportion correct as a function of prioritization type and status; for the LTM data, we used *d’* as a function of prioritization type, status, and whether the item had been tested in WM.

With this we calculated Bayes factors (BFs) representing the strength of evidence for a specified model (M1) or against a null or reduced model (M0). Additionally, evidence against a difference between an effect of interest can be calculated as well (BF_01_), where BF_01_ = 1/BF_10_. A BF_10_ > 1 suggests evidence *for* an effect, whereas a BF_10_ < 1 suggests evidence *against* an effect (i.e., evidence for the null hypothesis). BF_10_ = 10 indicates that the data are 10 times more likely under the alternative hypothesis than under the null hypothesis. Usually, BFs > 3 are regarded as providing substantial evidence for one hypothesis over the other (Wagenmakers, 2007). Unless stated otherwise, each BF reported estimates evidence for the effect (i.e., BF_10_). All BFs and Bayesian hierarchical linear models were estimated using the *BayesFactor* package in R with default settings and were run using 500,000 iterations. All data and analysis scripts can be accessed on OSF (https://osf.io/vyh5r/).

### Experiment 1

**Fig. 2:**
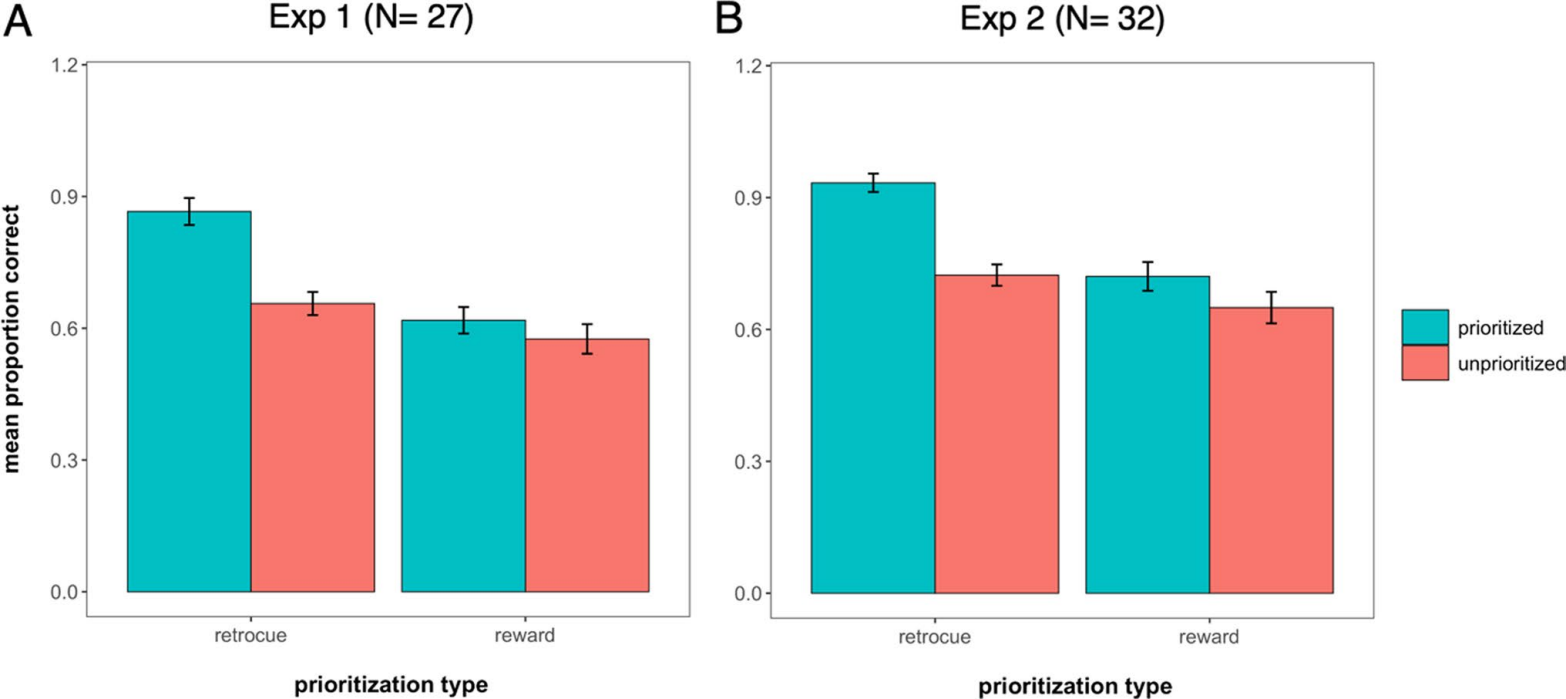
Working memory performance as a function of prioritization for Experiment 1 (**A**) and Experiment 2 (**B**). *Note.* Accuracy scores for prioritized and unprioritized items plotted as a function of prioritization condition. For the retro-cue condition, “prioritized” and “unprioritized” refer to cued and baseline items, respectively. For the reward condition, “prioritized” and “unprioritized” represent high-reward and low-reward items, respectively. Data and error bars indicate mean and within-subject 95% confidence intervals

The results of the WM test are illustrated in Fig. 2A. In WM, the fixed effects of interest were Prioritization Type (reward vs. retro-cue), Prioritization Status (prioritized vs. unprioritized), and their interaction. The Bayesian LME revealed evidence for an interaction of Prioritization Type × Prioritization Status (BF = 195), as well as both main effects of Prioritization Type (BF = 1.54 × 10^8^) and Prioritization Status (BF = 8.49 × 10^4^). Post hoc Bayesian one-sided *t* tests informed us of the effect of Prioritization Status separately for each Prioritization Type (better WM performance for prioritized information than for unprioritized information). There was strong evidence for a prioritization benefit given the retro-cue (BF = 1.21 × 10^7^), where recall of cued items was significantly greater than that of baseline items (no cues presented). However, there was no conclusive evidence for a prioritization benefit given the reward (BF = 1.26), suggesting that our reward prioritization manipulation did not have a credible impact on WM recall.

**Fig. 3:**
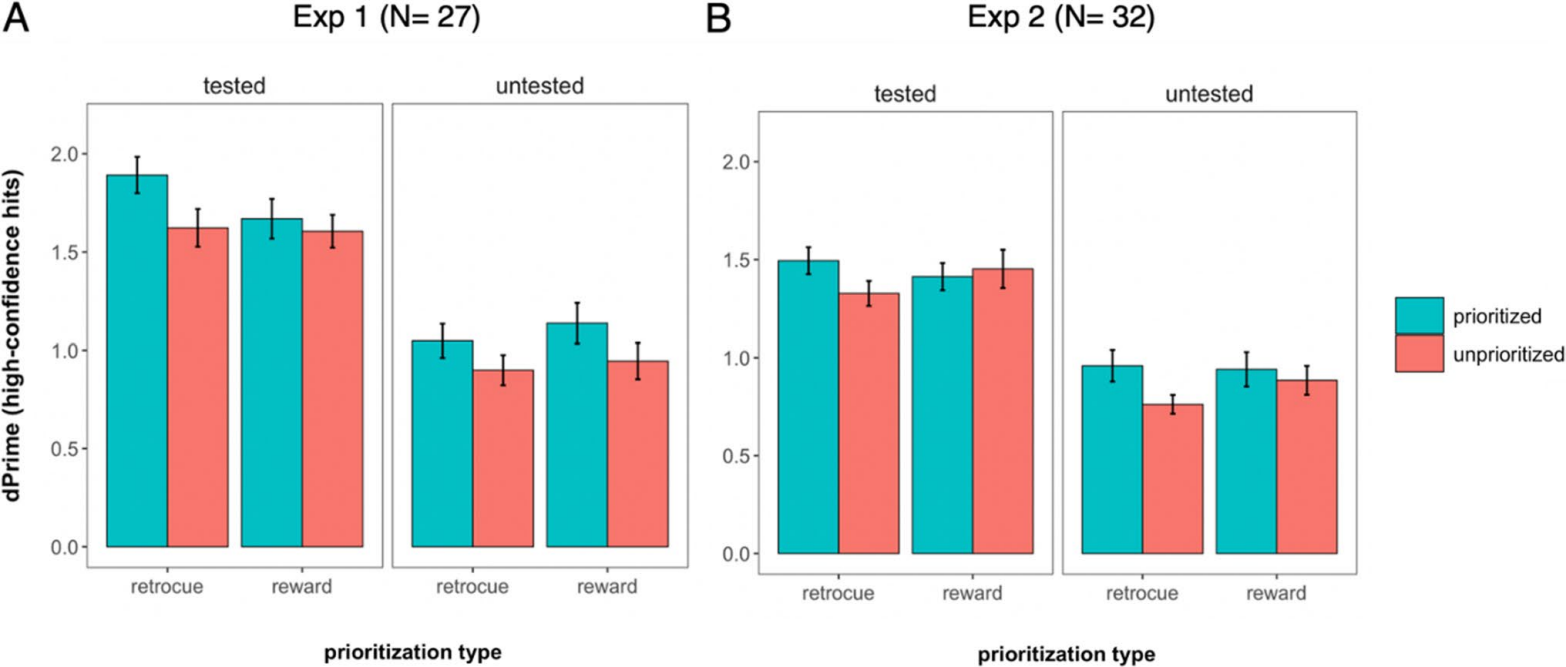
Long-term memory performance as a function of prioritization for Experiment 1 (**A**) and Experiment 2 (**B**)*. Note.* Computed *d′* scores for prioritized and unprioritized items plotted as a function of prioritization condition, split by WM test. For the retro-cue condition, “prioritized” represents performance for cued items and “unprioritized” includes the performance for both baseline (no cues presented) and noncued items from prioritization trials (in which another item was retro-cued). For the reward condition, “prioritized” and “unprioritized” represent high-reward and low-reward items, respectively. Data and error bars indicate mean and within-subject 95% confidence intervals

The results of the LTM test are illustrated in Fig. 3A. In LTM, the fixed effects of interest were Prioritization Type, Prioritization Status, and Tested Status (tested or untested in WM). The Bayesian LME revealed ambiguous evidence against the three-way interaction, Prioritization Type × Prioritization Status × Tested Status (BF_01_ = 1.60) and for the interaction of Tested Status × Prioritization Type (BF = 1.50), and substantial evidence against the interactions of Tested Status × Prioritization Status (BF_01_= 4.84) and Prioritization Type × Prioritization Status (BF_01_ = 3.39).

Next, we looked at the evidence for each main effect. There was strong evidence for main effects of Prioritization Status (BF = 86.5) and Tested Status (BF = 2.82 × 10^31^). However, there was conclusive evidence against a main effect of Prioritization Type (BF_01_ = 5.95). These results demonstrated that there was a prioritization benefit in LTM, but this effect was independent of the prioritization procedure used in WM (retro-cue or reward manipulations). Rather, the relevant factors determining LTM performance were whether an item had been in a prioritized or unprioritized state in WM and if it had been tested (which reflects the predicted, robust testing effect).

### Experiment 2

The results of the WM test are illustrated in Fig. 2B. In WM, like in Experiment 1, we found strong evidence for the interaction of Prioritization Type × Prioritization Status (BF = 40.16), as well as main effects of Prioritization Type (BF = 1.83 × 10^7^) and Prioritization Status (BF = 9.70 × 10^6^). Post hoc Bayesian one-sided *t* tests informed us of the effect of Prioritization Status separately for each Prioritization Type. There was evidence for a prioritization benefit given the retro-cue (BF = 9.91 × 10^9^) and given the reward (BF = 4.91). Therefore, the results of Experiment 2 show that with a more explicit and meaningful reward manipulation, there is a benefit of reward-based prioritization in WM. This subsequently allowed us to evaluate its consequences in LTM.

The results of the LTM test are illustrated in Fig. 3B. In LTM, as in Experiment 1, the fixed effects of interest were Prioritization Type, Prioritization Status, and Tested Status (tested or untested in WM). Bayesian LME models revealed that there was evidence against the three-way interaction, Prioritization Type × Prioritization Status × Tested Status (BF_01_ = 3.55), against Tested Status × Prioritization Status (BF_01_ = 3.85), and against Tested Status × Prioritization Type (BF_01_ = 5.22). There was anecdotal evidence for the interaction of Prioritization Type × Prioritization Status (BF = 2.51). For the main effects, there was substantial evidence for Prioritization Status (BF = 3.11) and Tested Status (BF = 6.87 × 10^30^), but evidence against Prioritization Type (BF_01_ = 4.59).

To examine the anecdotal interaction of Prioritization Type × Prioritization Status in LTM, we conducted post hoc comparisons of the expected effect of Prioritization Status separately for each Prioritization Type. These suggested strong evidence for a prioritization benefit for the retro-cue (BF = 1.40 × 10^3^), but substantial evidence *against* a prioritization benefit for the reward condition (BF_01_ = 6.00) in LTM.

### LTM analysis of retro-cue trials

**Fig. 4:**
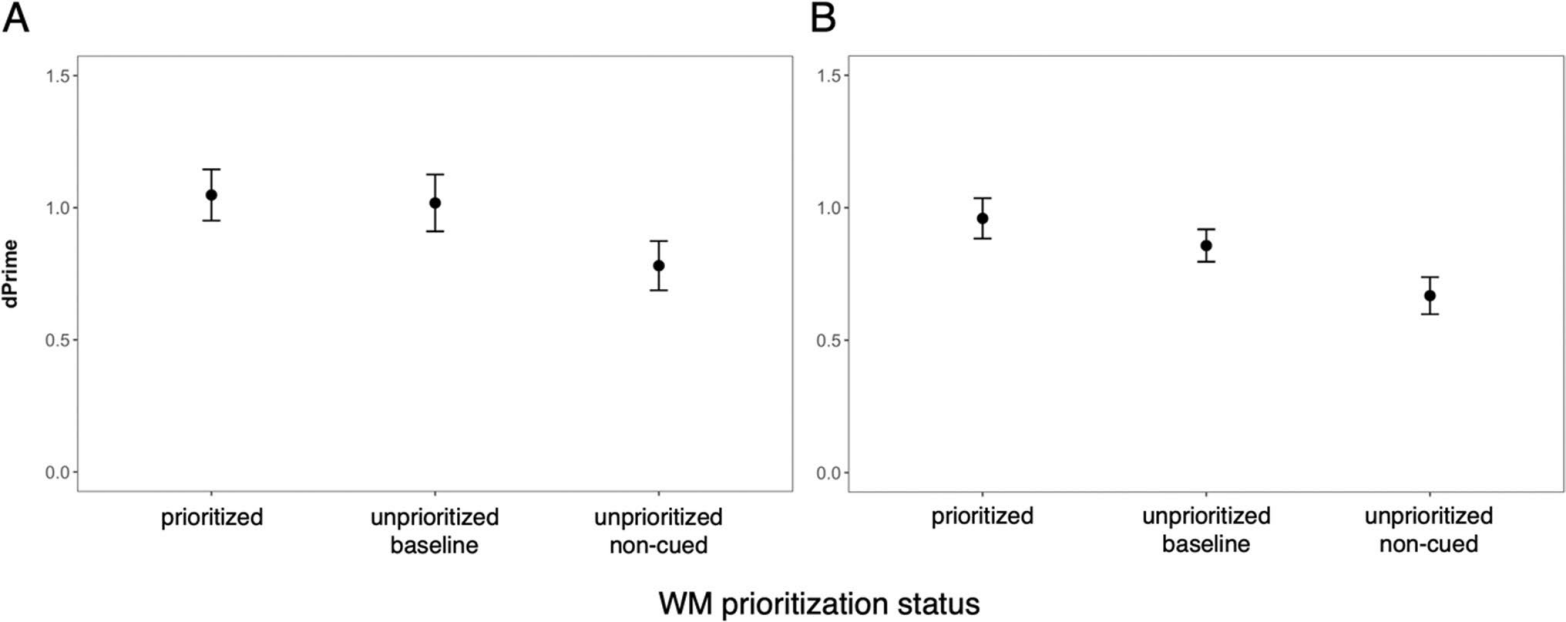
Long-term memory performance for retro-cue data as a function of WM prioritization status for Experiment 1 (**A**) and Experiment 2 (**B**). *Note.* Computed *d′* scores for untested items from retro-cue and baseline trials plotted as a function of WM status. LTM was compared for “prioritized” (cued items from prioritization trials), “unprioritized baseline” (noncued items from baseline trials), and “unprioritized noncued” (noncued items from prioritization trials in which another item was cued) items. Data and error bars indicate mean and within-subject 95% confidence intervals

To examine any potential differences in LTM performance across retro-cue and baseline trials, we zoomed further into LTM performance for the items that remained untested in the WM task to avoid any confound with the Tested Status factor (Fig. 4). The fixed effect of interest was WM Status (three levels; prioritized, unprioritized-baseline, unprioritized-noncued), for which we found evidence in both experiments (Exp 1: BF = 3.30, Exp 2: BF = 122).

As far as LTM performance is concerned, post hoc Bayesian one-sided *t* tests showed that (1) there is little to no evidence for a *benefit* of an item being prioritized in WM, compared with items in baseline trials in which none of the items were prioritized (BF_01_ = 2.58 and BF_01_ = 1.12 in Experiments 1 and 2, respectively); whereas (2) there is a *cost* associated for an item being unprioritized on trials where another item was cued to be prioritized, compared with items in baseline trials in which none of the items were prioritized (BF = 2.41 and BF = 9.92 in Experiments 1 and 2, respectively). There was also a cost for unprioritized noncued items when compared with the prioritized items from the same type of trial (BF = 6.29 and BF = 52.4 in Experiments 1 and 2, respectively).

## Discussion

This study has examined the effects of WM prioritization in both WM and LTM and is the first to directly compare cue- and reward-based retrospective prioritization procedures in this regard. Here, we presented participants with a WM task in which they prioritized previously presented information either by a retro-cue or a reward manipulation. We then compared the impact of both prioritization procedures in immediate and delayed memory within a single paradigm. Across both experiments, our results suggest that the type of prioritization procedure impacts the magnitude of its benefit in WM. Our results also suggest that WM prioritization generally affects LTM, and this seems to be more consistent when information was prioritized via a retro-cue rather than a reward pattern in WM.

In WM, we found that the type of prioritization procedure had an effect on how largely it benefited WM. In the first experiment, we observed a prioritization benefit, as has typically been observed in WM. This effect, however, was driven solely by the retro-cue manipulation as we did not observe meaningful evidence for a reward-based prioritization benefit in WM. We thus conducted a second experiment with a more explicit reward manipulation to better interpret any LTM effects. In the second experiment, we found a prioritization benefit across both manipulations in WM. Interestingly, the magnitude of the retro-cue benefit was nearly three times greater in magnitude than the reward benefit, as the percent increase of the prioritization benefit was 21.0% and 7.10% for the retro-cue and reward conditions, respectively. This suggests that the particular way in which attention is directed in WM modulates the size of the impact in WM—the boost being particularly enhanced with cue-based prioritization. Yet this comparison must be interpreted with caution as the validity of the priority signal is distinct in both prioritization procedures (i.e., how often the prioritized item will be tested; see Study limitations).

In LTM, we observed that prioritizing information in WM generally benefited LTM, supporting previous findings (Reaves et al., 2016; Sandry et al., 2020; Strunk et al., 2018). Like our findings in WM, our direct comparisons between cue- and reward-based prioritization hint at a difference between their effects in LTM. In particular, our study demonstrates that the overall LTM effect seems to be driven largely by cue-based WM prioritization, as we found evidence against a reward-based effect in LTM, despite having observed in Experiment 2 a reward-based benefit in WM. Interestingly, however, an exploratory analysis of untested (in WM) retro-cue data suggests that the LTM effect is driven by a *cost* to noncued (unprioritized) information, rather than a *benefit* of cued (prioritized) information.

### Is memory performance modulated by increased attention and maintenance?

Past research accounts have suggested that the more attentional resources are dedicated to behaviorally relevant information, the better the memory for that information in the short-term. This effect is supposedly driven by attentional processes and decreasing cognitive load in WM (Barrouillet et al., 2007; Souza et al., 2016; Vergauwe & Langerock, 2017), and in the long-term, by maintenance or elaboration processes (Bartsch et al., 2018; Forsberg et al., 2021; Loaiza & Souza, 2021). The overall effects of prioritization we observed at WM and LTM appear to support these accounts.

In particular, our data offer support for the long-term prioritization boost hypothesis, which assumes that attending more to information in WM strengthens its encoding in LTM. As explained in the introduction, the long-term prioritization boost hypothesis predicts better LTM performance for prioritized information than for unprioritized information, whereas the long-term prioritization impairment hypothesis predicts worse LTM performance for prioritized information than for unprioritized information. Our overall results appear to be in line with the predictions of the former. Moreover, this LTM effect was more pronounced for cue-based prioritization. This may indicate that LTM benefited more from a single, cued item receiving *all* focused attention (in the cue-based procedure), relative to the high-reward item receiving *more* focused attention than the other, low-reward items within a memory set (in the reward-based procedure).

Importantly, however, when examining the retro-cue data more closely,^7^ we uncovered that it was not the cued item that benefited from prioritization but rather the noncued items that suffered from not receiving any focused attention, leading to a cost in LTM performance. Thus, with respect to cue-based prioritization, we found an LTM cost for unprioritized items—but no LTM benefit for prioritized items—when compared with baseline items. When controlling for testing effects, this data suggest that the retro-cue effect observed in WM does not simply extend into LTM, as prioritized information was not remembered better than baseline items—yet there was a cost to remembering unprioritized information in LTM. As such, even though our LTM effects confirm the overall predictions of the long-term prioritization boost hypothesis, the results of the exploratory analysis are not in line with the notion that information that received focused attention in WM gets strengthened in LTM, since no boost or benefit was observed in LTM for cued information relative to noncued information (i.e., unprioritized information from trials without a cue). The unexpected observation that differences in LTM performance were driven by a cost of *un*prioritization suggests that the prioritization effect in LTM did not arise due to prioritized information being encoded more strongly into LTM through increased attentional focusing. Instead, it appears that the effect was due to reduced attentional focusing to unprioritized information which prevented or hindered its encoding into LTM. One way to explain this observation is through removal (Lewis-Peacock et al., 2018; Oberauer, 2001; Souza & Oberauer, 2016), whereby information that is no longer relevant for the task at hand is actively removed from WM. Active removal of information in WM has been shown to result in subsequent reduced accessibility of the information, hindering its recall from LTM (Dagry et al., 2017; Popov & Dames, 2023).

### Study limitations

In this study, we sought to compare the short- and long-term effects of two typical WM prioritization procedures. In doing so, we must acknowledge three aspects in which they differ: (1) their effects on WM load, (2) the validity of the priority signal indicating which item will be tested in WM, and (3) the meaningfulness of the cues for the participants. We detail them in the following.

Firstly, in the retro-cue paradigm, the cued item is the only item to reside inside the focus of attention, whereas noncued items can theoretically be dropped from WM entirely during WM maintenance, since prioritized items are always tested (i.e., 100% valid cue). In the reward-based paradigm, both high-reward and low-reward items theoretically reside inside the focus of attention, as the goal-relevant task demands require all items to remain accessible during WM maintenance. Therefore, based on the accounts previously stated, the more attention dedicated to information—as seen in the cue-based paradigm—the more WM and LTM will benefit from prioritization. This account thus explains the greater magnitude of the effect observed in WM and supports our explanation for the LTM effect being driven more so by cue-based prioritization. On this note, a recent study by (Atkinson et al., 2021), showed evidence that the reward-based prioritization boost for verbal memory was larger when verbal rehearsal was disrupted. Given that we did not instruct the use of articulatory suppression to minimize the use of verbal rehearsal of the familiar and nameable visual objects in our study, the use of rehearsal may be considered as one reason for the small reward benefit observed in our study. However, the same maintenance processes are likely to have contributed to performance in both cue- and reward-based conditions. Therefore, a differential involvement of verbal rehearsal is unlikely to explain the differences observed between the effects of both types of prioritization procedures.

Secondly, the comparison of reward and retro-cues in this study is further limited by differences in the validity of the priority signal. These differences are inherent to the procedures that have commonly been used. As our goal was to take a first step in directly comparing fairly typical prioritization procedures, this implied that we also were left with differences between these procedures regarding how often the prioritized item is to be tested at the end of a trial (the cued item has 100% probability of being tested, whereas the high-reward item has only 25% probability of being tested). In doing so, we established that both prioritization procedures do differ in their effect on memory performance. However, based on the data of the current study, we cannot determine whether the differences between retro-cue and reward manipulations of prioritization are due to differences in the underlying mechanisms involved in either manipulation, and/or due to differences in the validity of the priority signal used in both procedures. To disentangle the effects of priority signal validity and prioritization procedure, future studies should use 25% valid retro-cues, as a direct comparison to 1-1-1-4 reward cues.

Lastly, another issue concerning reward-based prioritization lies in the reward itself. The point values are arbitrarily associated with individual items and represent a rather weak manipulation of reward. One may circumvent this point by assigning actual monetary values in a reward-based prioritization paradigm, to test whether that would influence and perhaps strengthen the reward effect in WM and possibly LTM.

### Conclusion

The short- and long-term effects of WM prioritization have largely been studied separately using cue- and reward-based procedures to prioritize WM content. The current study is the first to *directly* compare both prioritization procedures in WM and LTM. By doing so, we have shown that the WM boost is larger and more consistent for cue-based WM prioritization. Our data also suggest that prioritizing information in WM *generally* enhanced LTM for that information, although the effect in LTM appeared to be driven predominantly by the retro-cue. Moreover, the effect of cue-based prioritization on LTM performance was found to be driven by a cost to unprioritized information, instead of a boost for the prioritized information. Future work should try to disentangle the relevance-based differences between WM prioritization procedures to better understand not only their short-term effects but also the drivers of long-term effects.

## Supplementary Information

ESM 1 (DOCX 33.7 kb)

## Footnotes

1 Posterior-maximal N2pc event-related potentials (ERPs) reflected spatial attentional bias during visual search.

2 This reward manipulation is different from the seminal value-based paradigm, where prioritized information was associated with a color, which was instructed to have a different value, rather than information being directly associated with different values.

3 A small number of participants (n = 5) participated in Experiment 1 from the University of Zurich.

4 The confidence ratings were used as a more sensitive measure of LTM performance. We were interested in collecting high-confidence responses to avoid participants from guessing and to control for familiarity effects (based on signal detection; see Yonelinas et al., 1996).

5 See Supplementary Table 1 for a complete breakdown of trials in the WM and LTM tasks.

6 These were the exact task instructions: “You will be able to accumulate these points for each correct response and win a small prize at the end of the experiment!”

7 Analysis of LTM performance for items from retro-cue and baseline trials in the WM task which were not probed in WM.
