# Supplementary material for: To be or not to be relevant: Comparing short- and long-term consequences across working memory prioritization procedures
Source: Atten Percept Psychophys. 2023 May 1;85(5):1486–98. doi: 10.3758/s13414-023-02706-4 (PMC10151114; doi:10.3758/s13414-023-02706-4)
Supplement: Supplementary file 1 — (DOCX 33.7 kb) [file 13414_2023_2706_MOESM1_ESM.docx]

**Supp. Table 1**

*Overview of Experimental Conditions and Trial Counts in WM and LTM Tasks*

|  |  |  |  | **WM** | | **LTM** | |
| --- | --- | --- | --- | --- | --- | --- | --- |
| **prioritization type** | | **prioritization status** | **WM status** | **# from WM** | **total in WM** | **total in LTM** | **# from WM** |
| **RETROCUE** | regular trials | P | tested | 32 | 32 | 32 | 16 |
|  | no-test trials |  | untested | 16 | 16 |  | 16 |
|  | no-test trials | UP | untested | 48 | 48 | 32 | 0 |
|  | regular trials |  | untested | 96 | 96 |  | 32 |
| BASELINE | | UP | tested | 32 | 128 | 32 | 16 |
|  |  |  | untested | 96 |  |  | 16 |
| **REWARD** | | P | tested | 16 | 64 | 32 | 16 |
|  |  |  | untested | 48 |  |  | 16 |
|  |  | UP | tested | 48 | 192 | 32 | 16 |
|  |  |  | untested | 144 |  |  | 16 |

|  | LEGEND |
| --- | --- |
| P | prioritized items in WM trials |
| UP | unprioritized items in WM trials |
| tested | tested items in WM trials |
| untested | untested items in WM trials |
| regular trials | retro-cue 100% valid trials |
| no-test trials | retro-cue no-test trials |
| baseline | retro-cue no-cue trials |
| reward | reward pattern trials |
